# Impact of an Educational Clinical Video Combined with Standard Helping Babies Breathe Training on Acquisition and Retention of Knowledge and Skills among Ethiopian Midwives

**DOI:** 10.3390/children10111782

**Published:** 2023-11-04

**Authors:** Amara J. Heard Stittum, Erika M. Edwards, Mahlet Abayneh, Asrat Demtse Gebremedhin, Delia Horn, Sara K. Berkelhamer, Danielle E. Y. Ehret

**Affiliations:** 1 Department of Pediatrics, Larner College of Medicine, University of Vermont, Burlington, VT 05401, USA; eedwards@vtoxford.org (E.M.E.); delia.horn@uvmhealth.org (D.H.); dehret@vtoxford.org (D.E.Y.E.); 2 Department of Mathematics and Statistics, College of Engineering and Mathematical Sciences, University of Vermont, Burlington, VT 05401, USA; 3 Vermont Oxford Network, Burlington, VT 05401, USA; 4 Department of Pediatrics and Child Health, St. Paul’s Hospital Millennium Medical College, Addis Ababa 1165, Ethiopia; mahlet.abayneh@sphmmc.edu.et; 5 Department of Pediatrics and Child Health, Addis Ababa University, Addis Ababa 1165, Ethiopia; asrat.dimtse@aau.edu.et; 6 Department of Pediatrics, University of Washington, Seattle, WA 98195, USA; berkelsa@uw.edu

**Keywords:** helping babies breathe (HBB), midwives, birth asphyxia, neonatal resuscitation, video, medical education

## Abstract

Helping Babies Breathe (HBB) is an evidence-based neonatal resuscitation program designed for implementation in low-resource settings. While HBB reduces rates of early neonatal mortality and stillbirth, maintenance of knowledge and skills remains a challenge. The extent to which the inclusion of educational clinical videos impacts learners’ knowledge and skills acquisition, and retention is largely unknown. We conducted a cluster-randomized controlled trial at two public teaching hospitals in Addis Ababa, Ethiopia. We randomized small training group clusters of 84 midwives to standard HBB vs. standard HBB training supplemented with exposure to an educational clinical video on newborn resuscitation. Midwives were followed over a 7-month time period and assessed on their knowledge and skills using standard HBB tools. When comparing the intervention to the control group, there was no difference in outcomes across all assessments, indicating that the addition of the video did not influence skill retention. Pass rates for both the control and intervention group on bag and mask skills remained low at 7 months despite frequent assessments. There is more to learn about the use of educational videos along with low-dose, high-frequency training and how it relates to retention of knowledge and skills in learners.

## 1. Introduction

While significant improvements in rates of neonatal mortality have occurred in highly resourced settings, a tremendous burden of neonatal death persists in poorly resourced settings, specifically low-income countries. A child is 10 times more likely to die in the first month of life if born in sub-Saharan Africa compared to a high-income country [1]. In Ethiopia specifically, under-5 mortality has declined by 60% from 2000 to 2016, while neonatal mortality has only declined by 41% during that same period, dropping from 49 deaths per 1000 live births to 29 deaths per 1000 live births [2].

Birth asphyxia is a leading cause of newborn mortality along with preterm birth and infections [1]. Helping Babies Breathe (HBB) is an evidence-based program that changed global education in newborn resuscitation [3] targeting the burden of morbidity and mortality due to birth asphyxia. HBB was launched in 2010, and since that time has been integrated into practice in healthcare facilities in over 80 low and middle income countries [4]. This program provides birth attendants with the skills needed to assist a newborn who does not breathe at birth. HBB utilizes interactive small-group learning and hands-on skills practice using a low-cost mannequin, a pictorial action plan, and a portable facilitator flip chart to teach birth attendants key skills needed to resuscitate a newborn who is not breathing [5]. Several studies have demonstrated that implementation of HBB improves newborn resuscitation and perinatal outcomes [6]. In a tertiary maternity hospital in Nepal, a quality improvement cycle following HBB implementation resulted in a reduction in intrapartum stillbirth and deaths of liveborn infants within the first hours by almost half [7]. Similarly, a study in Tanzania completed among three referral hospitals, four regional hospitals and one district hospital showed that implementation of HBB significantly reduced fresh stillbirths and early neonatal deaths [8]. A study in Sudan among 6 rural medical centers revealed a decline in fresh stillbirths (10.5 to 3.3 per 1000 births) as well as early neonatal deaths that occurred within one week of birth (13.5 to 4.3 per 1000 live births) when comparing cohorts before and after HBB training [9].

Despite studies demonstrating improvement in outcomes following implementation of HBB in a range of clinical settings, there are concerns for decline in knowledge and skills of providers over time. Researchers in Kenya showed that full mastery was not achieved without additional practice and continued learning [10]. They conducted HBB training in 71 facilities and found that in the follow up period, pass rates declined to 81% for resuscitation skill sets with a greater decline in skills than knowledge over time [10]. With this known decline, the use of digital interventions for retention of knowledge and skills acquired with HBB remains an active area of research and innovation [11].

Our study seeks to evaluate whether access to educational clinical videos might slow the known decline in knowledge and skills over time after initial training. Our objective was to assess midwives in Ethiopia over a 7-month time period comparing standard HBB training to standard HBB training supplemented with exposure to and ongoing access to an educational clinical video on newborn resuscitation. Knowledge and skills were tracked using standard HBB tools. Our hypothesis was that midwives exposed to standard HBB training supplemented with the educational clinical video would have less decline in their knowledge and skills following the initial training compared to midwives exposed to standard HBB training alone. 

## 2. Materials and Methods

We conducted a cluster-randomized trial at two public teaching hospitals in Addis Ababa, Ethiopia: Tikur Anbessa Hospital (TAH) and St. Paul’s Hospital Millennium Medical College (SPHMMC). Initial training was completed over a 5-day period from 8–12 November 2022 in Addis Ababa, Ethiopia and subsequent data were collected over 7 months and completed by 25 June 2023. Global Health Media Project (GHMP) gave permission to utilize their newborn resuscitation video for this study but was not involved in the proposal for study funding or the study design, implementation, analysis, and publishing of results. 

A cluster randomized design was deemed appropriate as the intervention was incorporated into the standard HBB training facilitated in small group sessions, mitigating the risk of cross contamination across individuals within training groups had they been individually randomized. Each HBB training small group was considered one cluster, with the intervention randomized at the level of the small group cluster. To calculate the number of clusters, we assumed a fixed number of clusters, ICC of 0.1538 based on the previous literature and fixed cluster sizes of six participants [12]. We utilized a type I error rate of 5% with 80% power to detect a simple difference of 1 point between two means for knowledge or skill. Adding the potential of a 15% loss to follow up, a total of 14 clusters (7 in each arm) of 6 participants each (84 total participants; 42 in each arm), were deemed adequate for the study.

An online randomization tool was utilized. The clusters were randomized into intervention and control arms in a ratio of 1:1. The participants, trainers and PI were unblinded on the day of the training. During the 7-month follow-up period, participant codes were utilized to link assessment scores to randomization clusters to minimize assessment bias.

Participants included midwives who were practicing at both of the two hospital facilities. This study focused on individuals who were relatively naïve to HBB training, therefore midwives who had undergone HBB training within the past year were excluded. Consent was not required since HBB training is a part of standard of care and this study only added a supplemental video. Prior to starting, the midwives were made aware of the study and that they would be assessed over a 7-month time period for additional follow-up.

The intervention was exposure to an educational video from GHMP titled *Teaching Points for Newborn Resuscitation.* This 15 min video demonstrated key concepts of newborn resuscitation as captured in actual footage of newborns in resource-limited areas undergoing resuscitation. Midwives who were randomized to this group watched the video at the start of their initial day of HBB training after completing the baseline assessments. Participants then received a full day standard HBB provider training course with an average ratio of 5 trainees per trainer, consistent with the control group experience. Following completion of training, participants in the intervention clusters were given access to the video via a digital messaging application for future reference. 

The control group clusters included midwives who received standard HBB training consisting of participation in a full day interactive HBB provider course with an average ratio of 5 trainees per trainer. Prior to starting enrollment, all trainers for both groups underwent HBB refresher training and practiced co-facilitation to ensure teaching methodologies were similar between groups. 

The primary outcome was results of the knowledge and skills assessments obtained in the immediate post-training period and at 1, 3, and 7 months after the initial training. Knowledge was assessed using standard HBB multiple choice knowledge check questions. Assessments for skills utilized the standard HBB program skills assessment tools including a bag-and-mask skill check and objective-structured clinical examinations (OSCE) A and B. 

On the day of initial enrollment, each midwife underwent a baseline assessment using the standard HBB knowledge check and bag-and-mask skill check prior to exposure to any educational content including the video for the intervention group. Following completion of training, they repeated the knowledge check and bag-and-mask skill check along with two separate OSCEs. The knowledge check, bag-and-mask skill check, and OSCEs were repeated at 1, 3 and 7 months through direct observation by one of the four trainers.

The data were collected by four experienced facilitators who had a background in education and HBB teaching as HBB master trainers. They completed the initial training and all subsequent follow-up assessments. Demographics regarding the midwives were collected along with feedback on preferred learning style.

We applied an intention-to-treat approach and compared the percent passing the knowledge check, bag-and-mask skill check, and OSCEs A and B between the control and intervention groups at each time interval. For the knowledge check, passing was defined as at least 15 of 18 questions correct. For the bag-and-mask skill check, passing was defined as successfully completing all 14 steps [11]. For OSCE-A, passing was defined as 9 of 12 correct as well as successfully completing the three required steps. For OSCE-B, passing was defined as 17 of 23 correct as well as successfully completing the five required steps [13]. The unit of analysis was at the level of the individual. We used chi-squared tests or Fisher’s exact tests when expected cell sizes were less than 5. To adjust for multiple comparisons, we applied a simple Bonferroni correction by dividing alpha, set at 0.05, by the number of comparisons (*n* = 18) and rounded down to be conservative, setting the *p*-value to determine statistical significance at <0.001. In addition to the mentioned assessments of knowledge and skills, we also evaluated changes over time to critical elements required for a passing score for OSCE A and B, as deemed by the HBB training course. All analyses were conducted in SAS 9.4.

## 3. Results

**Figure 1:**
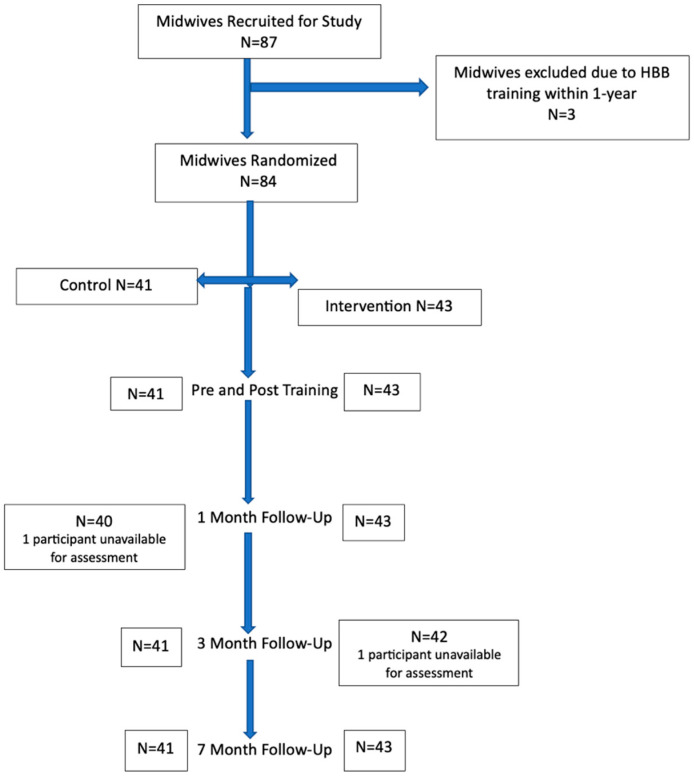
Consolidated Standards of Reporting Trials Diagram.

Out of the 87 participants, there were 44 from St Paul’s and 43 from Tikur Anbessa. We enrolled 84 midwives between the two academic institutions after initial exclusion (Figure 1).

Midwives in the two groups had comparable training and exposure (Table 1). Overall, 86.8% of the midwives in the control group and 88.3% in the intervention group had been practicing clinically for more than one year, yet 86.8% of the midwives in the control group and 90.7% of the midwives in the intervention group had never received formal HBB training. Despite this fact, 55.3% of the midwives in the control group and 47.5% of the midwives in the intervention group had participated in over 30 resuscitations as primary providers with 44.7% and 37.5% (control and intervention, respectively) reporting that they had provided bag and mask ventilation over 30 times.

Comparing both groups on individual assessment (Table 2), at baseline more participants passed the knowledge check in the intervention group (74.4%) than the control group (36.6%) (*p* < 0.0005). By the 7-month assessment, over 97% of participants in both groups passed the knowledge check. 

At baseline, neither group had any participants pass the bag-and-mask skill check, although both groups had immediate improvement during the post training period. At seven months, 51.2% of participants in the intervention group passed the bag-and-mask skill check compared to 41.9% in the control group (*p* < 0.39). There were no differences in pass rates for OSCE A between the two groups at any time point. At seven months, 72.1% of the intervention group and 73.2% of the control group passed the OSCE A (*p* < 0.92) while 79.1% of the intervention group and 75.6% of the control group passed the OSCE B (*p* < 0.71). Responses to the critical elements required for a passing score on OSCE A and B are shown in Table 3 and Table 4. One critical element had completion rates of less than 80% at various assessments over time: “stimulates breathing by rubbing the back”. This critical element is notable as it is required to pass both OSCE A and OSCE B. 

When we assessed participants at follow-up from both the control and intervention group, we inquired how often they had referenced materials. In the video group, 40.5% used a video, 23.8% used printed materials, 2.4% used simulation, and 33.3% used none. In the standard group, 17.1% used a video, 29.3% used printed materials, 0% used simulation, and 53.7% used none.

## 4. Discussion

Our study set out to determine if the addition of viewing and access to an educational clinical video on newborn resuscitation combined with standard HBB training would improve knowledge and skills retention over time compared to standard HBB training. We did not find a statistically significant difference between our intervention and control group on knowledge and skills post-training and at serial assessments over 7 months, indicating that the addition of the video did not impact skill retention. The midwives in our study, however, did not show the expected knowledge and skill loss over time as noted in the literature [9].

Resuscitation knowledge was assessed with the 18-item multiple choice HBB knowledge check. More than 90% of participants in the intervention group passed this assessment at each time point, showing maintenance of this knowledge over time with or without the video. Participants in the control group showed improvement in knowledge over time from their post-training assessment to the 7-month assessment. In contrast to findings by Bang et al. showing knowledge loss over time, our midwives showed maintenance or improvement in their knowledge. Although knowledge assessments were completed by administering the same 18-item multiple choice test at multiple time points, this methodology is similar among HBB studies assessing knowledge over time. 

Successfully completing all 14 steps of the bag-mask skill check was a challenge for the midwives in our study, with none of the midwives completing all steps on pre-assessment, and only 12% in the control group and 14% in the intervention group completing all 14 steps successfully on the post-assessment immediately after training. A study completed in Nagpur, India noted that midwives also found the bag-mask skill check difficult, ranked at level six out of six in difficulty prior to HBB training, and this ranking improved after training and refresher training to one out of six in difficulty in their study [14]. Although other HBB studies have found a peak in bag-mask skill scores post-training [11] followed by a loss in skill over time [10], our midwives exhibited continued growth in skills over time. It is notable, however, that although an increasing number of midwives in both the control and intervention groups passed the bag-mask skill check at each interval assessment, less than half of the midwives passed this skill check at 7 months.

While performance on bag-and-mask skills as a cohort improved, the midwives participating in this study did show skill loss over time on the OSCEs. OSCE-A includes preparation for birth, routine resuscitation including clearing the airway and stimulation by rubbing the infant’s back and placing the infant skin-to-skin with the mother and communicating. Participants had declining passing scores in OSCE-A from post-training to 7 months in both the control and intervention group. In OSCE-B, participants were required to use all the skills taught in the HBB course, including bag-mask ventilation within the Golden Minute, calling for help, taking steps to improve ventilation, checking the heart rate, and communicating the resuscitation and providing anticipatory guidance to the mother. Although there was not a significant difference in the scores between the control and intervention groups at any time period, the control group had an equivalent percent of participants passing at the post-training assessment and at 7 months but showed a decline in scores at the 1- and 3-month assessment. The intervention group had fewer participants passing on the post-training assessment, but this group with access to the newborn resuscitation teaching points clinical video showed continued improvement in the number of participants passing this OSCE at 1 and 3 months, which was maintained at 7 months. Similar studies which supplemented HBB training with video debriefing similarly showed improved rates of participants passing OSCEs [10,15]. While HBB implementation has been shown to reduce rates of fresh stillbirth and early neonatal mortality, the skills required for successful resuscitation can diminish over time [16].

Although educational clinical videos are now being embedded into remote teaching and facilitation of HBB with the adaptation of HBB via the World Health Organization’s Essential Newborn Care Course [17], the effectiveness of complementary clinical video on providers’ knowledge and skills and patient outcomes has not been thoroughly studied. This study, to our knowledge, is the first to look at the use of the newborn resuscitation teaching points video from GHMP as an educational tool for practicing midwives. A major strength of this study is the 100% follow-up rate of participants at the 7-month assessment, even with some midwives being lost to follow-up at the 1- and 3-month intervals. This study complements existing work using video for debriefing [15], and virtual reality simulation [11], all of which use integrated technology into the clinical learning environment. We targeted our participant population as practicing midwives with either no experience with HBB or having last taken a provider course more than a year ago. Although participants were relatively HBB-naive (86.8% of control participants and 90.7% of intervention participants without prior HBB training), the majority of midwives had been practicing clinically for more than a year and had experience being the primary provider in neonatal resuscitations and delivering bag-mask ventilation to neonates. Although the educational clinical video was easy to send to participants as a file on a cell phone messaging application, only a little under half mentioned at some point they referenced a video. It is possible that experienced midwives did not watch it again after the training as they felt their knowledge and skills were adequate and consistent with local expectations while students or pre-service trainees may have used the video more often as a reference for newly acquired skills. When we look at our two groups, 40.5% of the intervention group referenced a video compared to 17.1% of the control group. 

Several additional limitations are noted regarding this study. While we asked over time if the midwives had accessed any additional tools during follow-up assessments, we were unable to determine if it was the GHMP video or other videos being utilized. In addition, the frequency of viewing the video was not assessed. The video was intended for the intervention group alone but given that the midwives were randomized at the time of initial enrollment, it is possible that some in the control group were able to gain access to the video from their peers or downloaded it independently from the GHMP website. 

One explanation for the lack of overall knowledge and skill loss over time among the midwives in our study compared to prior publications is that the assessments at 1, 3 and 7 months after HBB training served as refresher training as all participants completed standard assessments and received feedback from HBB trainers regarding their performance. These assessments over a 7-month period, similar in both groups, could be viewed as an educational intervention. Therefore, it is unknown if there would be differences in overall passing scores or comparisons between the two groups at 7 months without an interval assessment at months 1 and 3. Our participants included midwives who work at two tertiary academic centers in the capital city of Ethiopia, which limits the generalizability of our results. Midwives who work in clinical practice in different levels of care within the health system, attend home births, or practice in rural areas might have different experiences and different approaches to maintaining clinical proficiency. Although all of our participants had access to mobile devices to view the GHMP video, it is possible that healthcare providers in other locations might not have reliable access to the internet or cell phone data plans to be able to download or view the video. Lastly, due to the sample size of our study, it is possible that there was a true difference between the control and intervention groups, but we were under-powered to detect it.

Although we did not find a difference between control and intervention groups, it is notable that the midwives in our study did not have the expected loss in knowledge and skill over time as seen in the literature, which may be due in part to the frequent reassessments with HBB trainers. Examining continued knowledge and skill assessment over time, in the context of refresher training, low-dose high-frequency skill practice, debriefing, and use of technology are areas worthy of further research. 

## 5. Conclusions

Healthcare providers are at risk of a decline in knowledge and skills over time following HBB training. The proportion of midwives passing standard HBB knowledge and skill assessments was not different immediately post-training or at 1, 3, or 7 months in the control group receiving standard HBB training compared to the intervention group with HBB training plus exposure to an educational clinical video. Frequent assessments by HBB master trainers over time may have contributed to the relative absence of knowledge and skill loss in participating midwives and is an important area for future research. 

## Figures and Tables

**Table 1:** Participant characteristics at baseline by control and intervention group.

	Control	Intervention
**How long have you been practicing as a midwife? (%)**	*n* = 38	*n* = 43
Less than six months Six months to 1 year 1 to 5 years More than 5 years	5.3 7.9 60.5 26.3	4.7 7.0 76.7 11.6
**Have you participated in HBB training in the past? (%)**	*n* = 38	*n* = 43
No, never Yes, more than one year ago	86.8 13.2	90.7 9.3
**How many deliveries (total) have you served as the primary provider for neonatal resuscitation? (%)**	*n* = 38	*n* = 40
Zero 1 to 10 11 to 20 21 to 30 More than 30	13.2 18.4 10.5 2.6 55.3	22.5 27.5 0.0 2.5 47.5
**How many times (total) have you provided bag-mask ventilation to a neonate at delivery? (%)**	*n* = 38	*n* = 40
Zero 1 to 10 11 to 20 21 to 30 More than 30	13.2 23.7 15.8 2.6 44.7	20.0 35.0 0.0 7.5 37.5
**Have you reviewed any materials in the last month as a “refresher” for knowledge or skills? (%)**	*n* = 38	*n* = 41
No Yes, printed HBB training materials Yes, educational video	68.4 15.8 15.8	61.0 9.8 29.3
**What is your preferred way of learning?**	*n* = 33	*n* = 42
Reading Listening Watching Practicing	12.1 3.0 6.1 78.8	4.8 0.0 23.8 71.4

**Table 2:** Comparison of percent of participants who passed in the control and intervention groups by type of assessment and time point.

	Control		Intervention		*p*-Value
	N	%	N	%	
**Knowledge Check**					
Pre	41	36.6	43	74.4	0.0005
Post	41	78.1	43	90.7	0.11
One Month	40	80.0	43	90.7	0.17
Three Month	41	85.4	42	92.9	0.32
Seven Month	41	97.6	43	97.7	1.0
**Bag-Mask Skill Check**					
Pre	41	0.0	43	0.0	NA
Post	41	12.2	42	14.3	0.78
One Month	41	17.1	42	19.1	0.82
Three Month	41	14.6	42	23.8	0.29
Seven Month	41	51.2	43	41.9	0.39
**OSCE-A**					
Post	40	85.0	43	90.7	0.52
One Month	40	75.0	43	96.1	0.21
Three Month	41	82.9	42	88.1	0.50
Seven Month	41	73.2	43	72.1	0.92
**OSCE-B**					
Post	39	74.4	43	67.4	0.50
One Month	40	55.0	43	69.8	0.17
Three Month	40	67.5	41	80.5	0.19
Seven Month	41	75.6	43	79.1	0.71

NA = Not Applicable.

**Table 3:** Percent correct on OSCE-A required questions in the control and intervention groups by type of assessment and time point.

	Control		Intervention	
**Prepares an area for ventilation and checks function of bag, mask and suction device**	N	%	N	%
Post	40	100.0	43	97.7
One Month	40	95.0	44	100.0
Three Month	41	92.7	42	97.6
Seven Month	41	90.3	43	95.4
**Dries thoroughly**				
Post	40	95.0	43	97.7
One Month	40	97.5	43	95.4
Three Month	41	100.0	42	97.7
Seven Month	41	100.0	43	100.0
**Stimulates breathing by rubbing the back**				
Post	40	92.5	43	97.7
One Month	40	82.5	43	90.7
Three Month	41	87.8	42	95.3
Seven Month	41	82.9	43	79.1

**Table 4:** Percent correct on OSCE-B required questions in the control and intervention groups by type of assessment and time point.

	Control		Intervention	
**Stimulates breathing by rubbing the back**	N	%	N	%
Post	39	89.7	43	90.7
One Month	40	77.5	43	76.7
Three Month	40	87.5	41	85.4
Seven Month	41	95.1	43	83.7
**Achieves a firm seal as demonstrated by chest movement**				
Post	39	92.3	43	90.7
One Month	40	87.5	43	97.7
Three Month	40	92.5	41	100.0
Seven Month	41	97.7	43	100.0
**Ventilates at 40 breaths/minute (30–50 acceptable)**				
Post	39	94.9	43	88.4
One Month	40	90.0	43	90.7
Three Month	40	87.5	41	95.1
Seven Month	41	87.8	43	95.4
**Evaluates for breathing or chest movement**				
Post	39	100.0	37	86.1
One Month	40	80.0	43	95.4
Three Month	40	97.5	41	100.0
Seven Month	41	95.1	43	95.4
**Reapplies mask**				
Post	39	92.3	43	100.0
One Month	40	100.0	43	100.0
Three Month	40	97.5	41	97.6
Seven Month	41	97.6	43	100.0
**Repositions head**				
Post	39	94.9	43	100.0
One Month	40	95.0	43	97.7
Three Month	40	100.0	41	97.6
Seven Month	41	95.1	43	100.0

## Data Availability

The data presented in this study are available on request from the corresponding author.
